# A key metabolic integrator, coenzyme A, modulates the activity of peroxiredoxin 5 via covalent modification

**DOI:** 10.1007/s11010-019-03593-w

**Published:** 2019-08-02

**Authors:** Jovana Baković, Bess Yi Kun Yu, Daniel Silva, Sew Peak Chew, Sangeun Kim, Sun-Hee Ahn, Laura Palmer, Lujain Aloum, Giacomo Stanzani, Oksana Malanchuk, Michael R. Duchen, Mervyn Singer, Valeriy Filonenko, Tae-Hoon Lee, Mark Skehel, Ivan Gout

**Affiliations:** 1 Department of Structural and Molecular Biology, University College London, London, WC1E 6BT UK; 2 Biological Mass Spectrometry & Proteomics Cell Biology, MRC Laboratory of Molecular Biology, Cambridge, CB2 0QH UK; 3 Department of Oral Biochemistry and Department of Molecular Medicine (BK21plus), Dental Science Research Institute, School of Dentistry, Chonnam National University, Gwangju, Republic of Korea; 4 Department of Cell and Developmental Biology, University College London, London, WC1E 6BT UK; 5 Bloomsbury Institute of Intensive Care Medicine, University College London, London, WC1E 6BT UK; 6 Institute of Molecular Biology and Genetics, National Academy of Sciences of Ukraine, Kyiv, 03680 Ukraine

**Keywords:** Peroxiredoxin 5 (Prdx5), Coenzyme A (CoA), Oxidative stress, Redox regulation, Reactive oxygen species (ROS)

## Abstract

Peroxiredoxins (Prdxs) are antioxidant enzymes that catalyse the breakdown of peroxides and regulate redox activity in the cell. Peroxiredoxin 5 (Prdx5) is a unique member of Prdxs, which displays a wider subcellular distribution and substrate specificity and exhibits a different catalytic mechanism when compared to other members of the family. Here, the role of a key metabolic integrator coenzyme A (CoA) in modulating the activity of Prdx5 was investigated. We report for the first time a novel mode of Prdx5 regulation mediated via covalent and reversible attachment of CoA (CoAlation) in cellular response to oxidative and metabolic stress. The site of CoAlation in endogenous Prdx5 was mapped by mass spectrometry to peroxidatic cysteine 48. By employing an in vitro CoAlation assay, we showed that Prdx5 peroxidase activity is inhibited by covalent interaction with CoA in a dithiothreitol-sensitive manner. Collectively, these results reveal that human Prdx5 is a substrate for CoAlation in vitro and in vivo, and provide new insight into metabolic control of redox status in mammalian cells.

## Introduction

Peroxiredoxins (Prdxs) are antioxidant enzymes that neutralise a broad range of reactive oxygen and nitrogen species, including H_2_O_2_, alkyl hydroperoxides and peroxynitrites. Prdxs are highly conserved throughout evolution and broadly distributed from bacteria to humans [1, 2]. The family of mammalian Prdxs can be subdivided into three major subclasses based on sequence, structural homology and catalytic mechanism: typical 2-cysteine (2-Cys) Prdxs (Prdxs1-4), atypical 2-Cys Prdx (Prdx5) and 1-Cys Prdx (Prdx6). Prdxs exhibit different subcellular localisation, regulation and substrate specificity. All members of the Prdx family share a common mechanism of peroxide-reducing activity that involves oxidation of a conserved peroxidatic cysteine (CysP) to a sulphenic acid (R-SOH), but differ in the mode of its reduction back to a thiol. The activity and cellular functions of Prdxs are tightly controlled by regulatory interactions and post-translational modifications. A diverse range of binding partners for Prdxs have been identified, including proteins involved in signal transduction, metabolic processes and antioxidant defence [3–5]. Reversible thiol modifications, including S-sulphenylation, S-sulphinylation, S-glutathionylation and S-nitrosylation, as well as phosphorylation and acetylation were shown to play key roles in regulating peroxidase activity, subcellular localisation and redox signalling of Prdx family members [3–6]. By regulating intracellular peroxide levels, Prdxs were shown to participate in the regulation of cell proliferation, differentiation and redox signalling under physiological and pathological conditions [1, 7, 8].

Prdx5 is the most divergent isoform among mammalian peroxiredoxins and shows a surprisingly wide subcellular distribution, including the mitochondria, peroxisomes, nucleus and cytoplasm [6]. The catalytic mechanism of Prdx5 is initiated by the reduction of a peroxide substrate, during which the peroxidatic Cys48 thiol (Cys48-SH) is oxidised to sulphenic acid (Cys48-SOH). The resolving Cys152 then reacts with Cys48-SOH to form an intramolecular disulphide. Due to the large distance between Cys152-SH and Cys48-SOH, the enzyme undergoes a significant conformational change to allow the two groups to come into proximity. Thioredoxins (Trx1 in the cytosol or Trx2 in the mitochondria) reduce the disulphide bond (Cys48-SS-Cys152) and regenerate a sulfhydryl group on Cys48, thereby reactivating the enzyme. This reaction requires the reductant NADPH, which is used by thioredoxin reductase (TrxR) to regenerate Trxs.

Consistent with its antioxidant properties, the knockdown of Prdx5 makes cells more susceptible to apoptosis induced by oxidative stress, while its overexpression prevents programmed cell death [9]. Transcriptional or translational upregulation or downregulation of the Prdx5 gene was shown to take place in response to various stresses and during pathophysiological conditions such as cancer [7].

Coenzyme A (CoA) is an obligatory cofactor in all living cells synthesised from pantothenate (Vitamin B5), adenosine triphosphate (ATP) and cysteine [10]. CoA and its thioester derivatives (acetyl CoA, malonyl CoA and succinyl CoA among many others) are key players in major catabolic and anabolic pathways and the regulation of gene expression [10–13]. Many human pathologies, including cancer, diabetes and neurodegeneration, have been associated with abnormal biosynthesis and homeostasis of CoA and its derivatives [14–16].

We have recently discovered a novel non-canonical function of CoA in redox regulation, involving covalent attachment of this coenzyme to cellular proteins in response to oxidative and metabolic stress, termed protein CoAlation. The development of new research tools and methodologies has been instrumental in uncovering protein CoAlation as a widespread post-translational modification and revealing its role in redox regulation. These developments afford us the unique opportunity to readily detect protein CoAlation in eukaryotic and prokaryotic cells in response to oxidative and metabolic stress and identify CoAlated proteins. To date, nearly one thousand proteins have been found to be CoAlated in bacteria, mammalian cells and tissues in response to oxidising agents and metabolic stress [17, 18].

Here, we report for the first time that Prdx5 is covalently modified by CoA when cells and tissues are exposed to oxidative and metabolic stress. Mass spectrometry analysis revealed that the peroxidatic Cys48 is CoAlated in cellular response to oxidative stress. We demonstrated by employing an in vitro CoAlation assay that covalent binding of CoA to Prdx5 results in complete inhibition of its peroxidase activity, which is reversed by reduction with DTT. Based on these findings, we propose that covalent modification of peroxidatic cysteine 48 in Prdx5 in response to oxidative and metabolic stress not only protects it from overoxidation but may function as regulatory mechanism of redox signalling.

## Materials and methods

All common chemicals were obtained from Sigma–Aldrich unless otherwise stated. The generation and characterisation of the anti-CoA antibody (1F10) was described previously [19]. Anti-Prdx5 rabbit antibody were obtained from Abcam and secondary Alexa Fluor 680 goat polyclonal anti-mouse IgG and Alexa Fluor 800 goat polyclonal anti-rabbit IgG were from Life Technologies.

### Cell culture

HEK293/Pank1β cells were generated as previously described [17]. Cells were maintained in DMEM (Lonza) supplemented with 10% foetal bovine serum (FBS, Hyclone) and 10 ml/L Penicillin–streptomycin (P/S, Lonza).

### Cardiomyocytes preparation

Cardiomyocytes were isolated from adult male Wistar rats weighing 300–350 g. Primary cardiomyocytes were isolated as previously described with minor changes [20]. The isolated cardiomyocytes were suspended in M199 media (ZenBio) supplemented with 5 mmol/l creatine, 5 mmol/l taurine, 2 mmol/l carnitine, 100 IU/ml penicillin and 100 IU/ml streptomycin, then plated on 6 cm dishes (VWR UK) pre-coated with 5 mcg/ml laminin. Cells were plated at a density of 340.000 rod-shaped cells per dish and incubated overnight at 37 °C and 5% CO_2_ before experiments the following day. > 90% of attached cells appeared to be rod-shaped and non-contracting.

### Plasmids and transient transfection

Human Prdx5 cDNA without the signal sequence was cloned into the pCR3.1/Prdx5 vector as previously described [21]. HEK293/Pank1β cells were transfected at ~ 60% confluence with pCR3.1-MAR plasmid vectors encoding streptavidin binding protein (SBP); SBP-tagged full-length WT-hPrdx5, hPrdx5C48S, hPrdx5C152S and hPrdx5C48/152S. Cells were transfected according to the manufacturer’s protocol using Turbofect reagent (Thermo Scientific).

### Oxidative and metabolic stress induction

Following transient transfection or an overnight sub-culturing, cells were incubated for 18 h in pyruvate-free DMEM supplemented with 5 mM glucose, 10% FBS and 10 ml/L P/S. Pyruvate was removed from the media because it can act as an antioxidant and inactivate ROS. Cells were then treated with or without H_2_O_2_ (500 μM), diamide (500 μM) or menadione (50 μM), and incubated for 30 min at 37 °C. To induce metabolic stress, the medium of transiently transfected cells was replaced with pyruvate- and glucose-free DMEM and cells were incubated for an additional 18 h. To allow recovery, full DMEM media was re-introduced after 18 h and cells were incubated in at 37 °C for 30 min.

### Cell lysis, immunoprecipitation and western blot analysis

Harvested cells were lysed for 20 min in lysis buffer (at 4 °C) containing 50 mM Tris–HCl pH 7.5, 150 mM NaCl, 5 mM ethylenediaminetetraacetic acid (EDTA), 50 mM sodium fluoride (NaF), 5 mM tetra-sodium pyrophosphate (Na_4_P_2_O_7_) and 1% Triton X-100, supplemented with fresh 100 mM NEM and fresh 1 × Protease Inhibitor Cocktail (PIC, Roche). Total cell lysates were centrifuged at 20,817×*g* for 10 min at 4 °C and the supernatant was collected for further analysis. Protein concentration was measured using the Bicinchoninic acid Protein Assay Kit (Thermo Scientific). Immunoprecipitation of endogenous Prdx5 from cell lysates was carried out using Protein G Sepharose (Generon) and anti-Prdx5 antibody. Affinity purification of SBP-Prdx5 was carried out using streptavidin beads (Upstate Biotechnology Inc). Proteins were eluted with 2× SDS loading buffer and analysed by anti-CoA Western blots. SDS-PAGE separated proteins were transferred to a PVDF membrane (Bio-Rad Laboratories) which was then blocked with Odyssey blocking buffer. The membrane was incubated in primary antibodies for 2 h at room temperature (RT) or overnight at 4 °C, and with secondary antibodies for 30 min RT. Immunoreactive bands were visualised using Odyssey Scanner CLx and Image Studio Lite software (LI-COR Biosciences).

### Heart perfusion

Sprague–Dawley rats (120–300 g) were used in this study. All experiments involving animals were performed in accordance with the European Convention for the Protection of Vertebrate Animals used for Experimental and Other Scientific Purposes (CETS no. 123) and the UK Animals (Scientific Procedures) Act 1986 amendment regulations 2012. Heart perfusion was performed as previously described, with minor modifications. BSA was omitted and 11 mM glucose and 1.8 mM CaCl_2_ were added to Krebs–Henseleit buffer (KHB) [22]. Hearts were perfused with KHB for 10 min followed by 20 min perfusion in the presence or absence of 100 µM H_2_O_2_.

### Expression and affinity purification of Prdx5

*Escherichia coli* BLR (DE3) cells were transformed with plasmid containing His-tagged human Prdx5 coding sequence [21]. Expression and affinity purification of His-Prdx5 on Talon Resin (Clontech Laboratories) was performed as described previously [23]. Eluted protein was dialysed against 20 mM Tris–HCl (pH 7.5) containing 1 mM EDTA and stored at − 80 °C.

### In vitro CoAlation assay

Purified recombinant His-Prdx5 (0.5 µg) was incubated with a mixture of oxidised and reduced forms of CoA (CoASH and CoASSCoA, 1 mM final) in 20 mM Tris–HCl, pH 7.5 for 30 min at RT. The mixture was passed through a BioSpin 6 column (Bio-Rad) to remove excess CoA, and this preparation of Prdx5 was further used in activity assays. For Western blot analysis, NEM (10 mM final) was added to the samples for 10 min before mixing with SDS loading buffer (1× final) with or without DTT.

For SDS-PAGE analysis, 2 µg of His-Prdx5 was incubated with CoA and CoASSCoA as previously described, or with H_2_O_2_ (1 mM final) for 10 min or with buffer alone, and was mixed with reducing or non-reducing loading buffer, before loading on the gel.

### Prdx5 activity assay

Prdx5 activity was measured using the thioredoxin system as described previously [24]. The rate of H_2_O_2_ degradation was measured by monitoring the decrease in *A*_340_ caused by NADPH oxidation. The assay was performed in a 150 μl reaction mixture, containing 50 mM Hepes–NaOH (pH 7), 200 μM NADPH, 760 nM mouse TrxR, 11 μM human Trx and 1 μM Prdx5 (control or in vitro CoAlated). The mix was incubated at 37 °C for 5 min, and the reaction initiated by the addition of 500 μM H_2_O_2_.

### Statistical analysis

Where appropriate, values are given as means ± SEM.

### Mass spectrometry and data processing

Peptides resulting from enzymatic digestion were analysed by nano-scale capillary LC–MS/MS using an Ultimate U3000 UPLC System (Dionex) fitted with a 100 µm × 2 cm PepMap100 C_18_ nano trap column and a 75 μm × 25 cm PepMap100 C_18_ nano analytical column (Dionex). Peptides were eluted using an acetonitrile gradient and sprayed directly via a nano-flow electrospray ionisation source into the mass spectrometer (Orbitrap Velos, Thermo Scientific). The mass spectrometer was operated in data dependent mode, using a full scan (m/z = 350–1600) in the Orbitrap analyser, with a resolution of 60,000 at m/z = 400, followed by MS/MS acquisitions of the 20 most intense ions in the LTQ Velos ion trap. Maximum FTMS scan accumulation times were set at 250 ms and maximum ion trap MSn scan accumulation times were set at 200 ms. The Orbitrap measurements were internally calibrated using the lock mass of polydimethylcyclosiloxane at m/z 445.120025. Dynamic exclusion was set for 30 s with exclusion list of 500. LC–MS/MS raw data files were processed as standard samples using MaxQuant [25] version 1.5.2.8 which incorporates the Andromeda search engine. MaxQuant processed data were searched against either a Rat or Human UniProt protein database. Carbamidomethyl cysteine, Acetyl N-terminal, N-ethylmaleimide cysteine, oxidation of methionines, CoAlation of cysteine with delta mass 338, 356 and 765, were set as variable modifications. For all data sets, the default parameters in MaxQuant were used, except MS/MS tolerance, which was set at 0.6 Da and the second peptide ID was unselected.

## Results

### Mass spectrometry-based identification of Prdx5 CoAlation

**Fig. 1:**
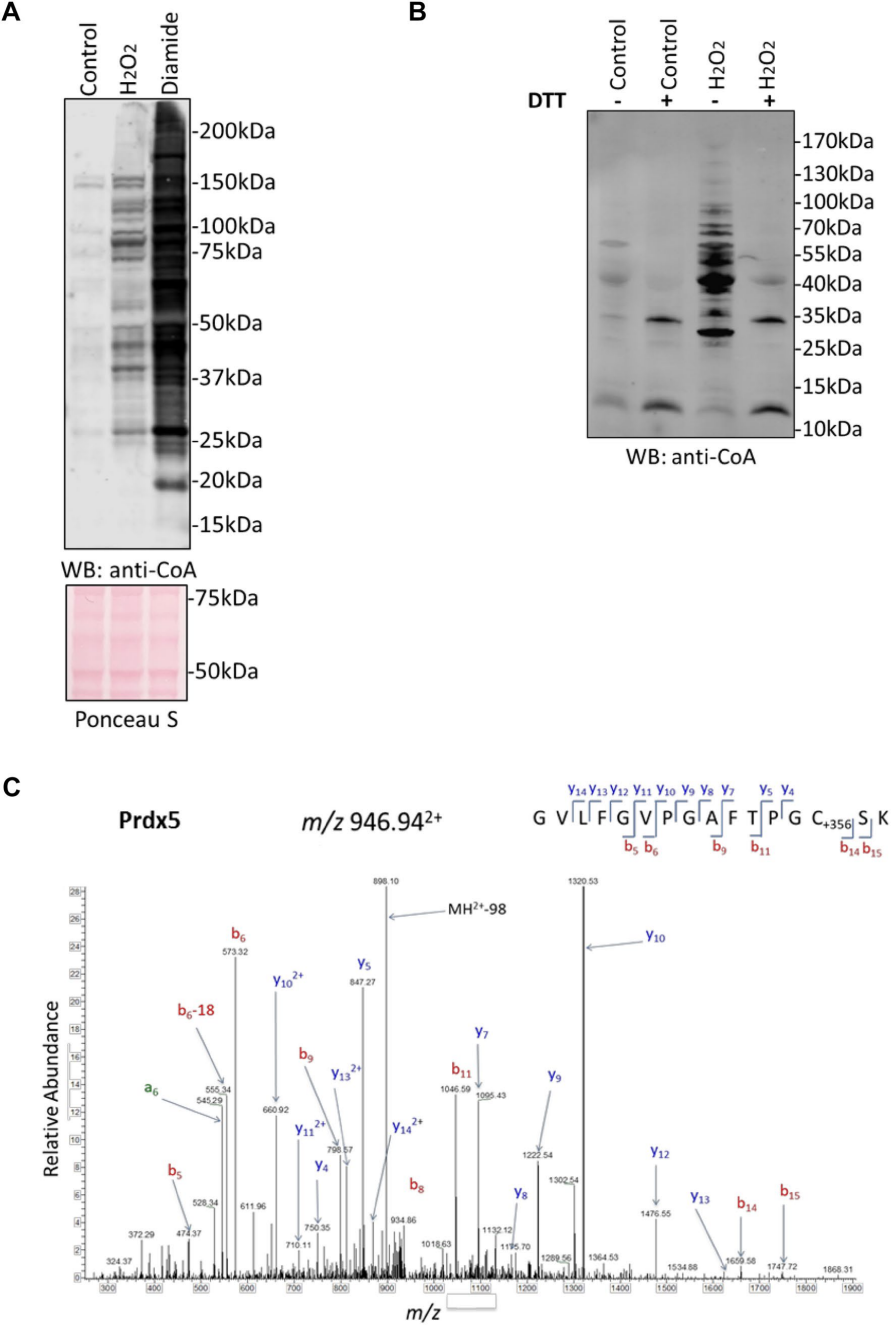
Prdx5 is CoAlated in HEK293/Pank1β cells and in perfused rat heart in response to oxidative stress. **a** Anti-CoA Western blot reveals extensive modification of cellular proteins by CoA in HEK293/Pank1β cells treated with 500 µM H_2_O_2_ and 500 µM diamide for 30 min. **b** Analysis of protein CoAlation in isolated rat hearts perfused in the presence or absence of 100 µM H_2_O_2_ for 20 min, N = 3. **c** LC–MS/MS spectrum of a CoAlated peptide (GVLFGVPGAFTPGCSK), corresponding to peroxiredoxin 5 (Prdx5). The Prdx5-derived peptide was immunoprecipitated with anti-CoA antibody 1F10 from trypsin/LysC digested protein extracts of rat heart perfused with 100 µM H_2_O_2_ and analysed by LC–MS/MS as previously described (Y. Tsuchiya et al.)

To study the role of CoA in redox regulation in mammalian cell and tissues, we employed the HEK293/Pank1β cell and Langendorff-perfused heart models. HEK293/Pank1β cells were grown to 60% confluency in complete Dulbecco’s modified Eagle’s medium (DMEM) and then cultured in pyruvate-free, low glucose DMEM for 18 h. Oxidative stress was induced by incubating cells in the presence of 500 µM H_2_O_2_ or 500 µM diamide for 30 min. Protein extracts were separated on non-reducing SDS-PAGE and immunoblotted with anti-CoA monoclonal antibody. As shown in Fig. 1a, only a few proteins are CoAlated at a background level in control non-treated cells, while extensive protein CoAlation is observed in response to H_2_O_2_ and diamide.

In addition, we used the Langendorff-perfused heart model for examining protein CoAlation in rat heart exposed to oxidative stress. In this study, isolated rat hearts were perfused with 100 µM H_2_O_2_ for 20 min and homogenised as described in Materials and Methods. We found that H_2_O_2_ perfusion significantly increased protein CoAlation when compared to control, and this was reversed to the level of untreated control by DTT (Fig. 1b).

Extensive protein CoAlation in response to oxidative stress in HEK293/Pank1β and perfused rat heart prompted us to identify CoA-modified proteins using the developed methodology [17]. In our studies, we used HEK293/Pank1β cells which overexpress pantothenate kinase 1β (the main rate-limiting enzyme in CoA biosynthesis) and produce comparable levels of CoA as those found in rat heart, liver, kidney or primary cardiomyocytes. In fact, analysis showed that established cell lines HepG2 and HEK293 contain much lower concentrations of CoA when compared to those found in the previously mentioned sources [26]. A diverse range of proteins was found to be CoAlated in prepared samples from HEK293/Pank1β cells and perfused rat heart. The vast majority of CoAlated proteins were found to be involved in metabolic processes (over 65%), as well as proteins implicated in stress response. Notably, an atypical member of the peroxiredoxin family, Prdx5, was found to be CoAlated at peroxidatic Cys48 in both HEK293/Pank1β cells and perfused rat heart exposed to H_2_O_2_. Figure 1c shows the liquid chromatography tandem-mass spectrometry spectrum of a cysteine-containing peptide derived from Prdx5 (GVLFGVPGAFTPGCSK) with an increase in 356 Da, corresponding to covalently attached 4-phosphopantetheine to cysteine residue.

### Prdx5 CoAlation is induced in cells by a panel of oxidising agents

**Fig. 2:**
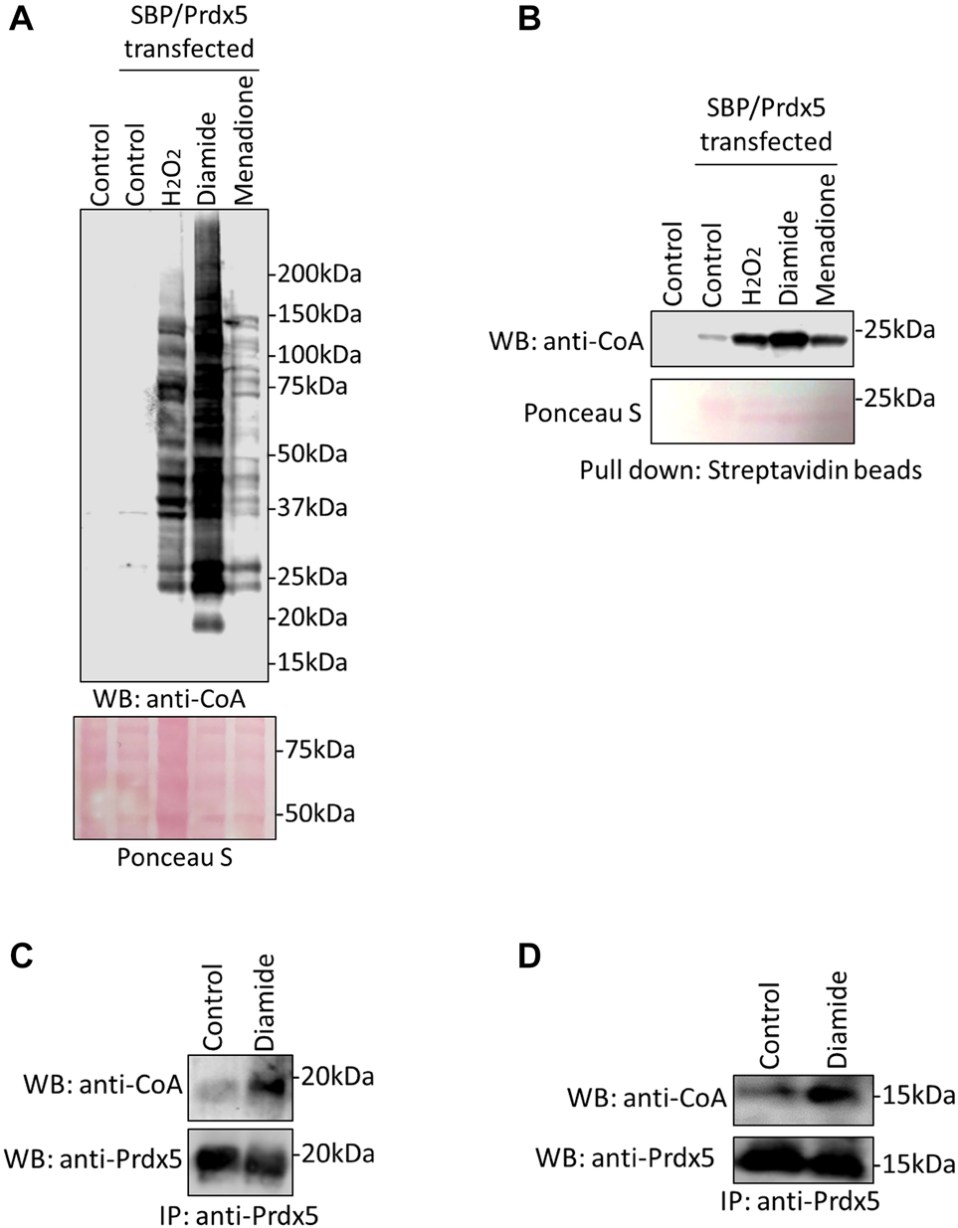
CoAlation of endogenous and transiently overexpressed Prdx5 is induced in response to oxidising agents. **a** Western blot analysis of protein CoAlation in HEK293/Pank1β cells treated for 30 min with diamide (500 µM), menadione (50 µM) or H_2_O_2_ (500 µM). **b** Transiently overexpressed SBP-tagged Prdx5 was pulled down from cell lysates and bound proteins examined by immunoblotting with anti-CoA antibodies. **c** Western blot analysis of immunoprecipitated endogenous Prdx5 from HEK293/Pank1β cells or primary cardiomyocytes **d** treated or not treated with 500 µM diamide for 30 min. N = 3

Induction of Prdx5 CoAlation by H_2_O_2_ in both experimental models prompted us to examine whether Prdx5 is CoAlated in cellular response to other oxidising agents. In this study, cells were transfected with pCR3.1-MAR/wt-Prdx5 plasmid, which drives the expression of human Prdx5 with the N-terminally tagged streptavidin binding peptide (SBP). Transfected cells were grown in pyruvate-free and low glucose media for 18 h and then treated with or without 500 µM H_2_O_2_, 500 µM diamide or 50 µM menadione for 30 min to induce oxidative stress. Transiently overexpressed SBP-Prdx5 was pulled down from cell lysates using streptavidin beads and both total and the pulled down proteins were analysed by SDS-PAGE under non-reducing conditions followed by immunoblotting with anti-CoA antibody. As shown in Fig. 2a, treatment of cells with H_2_O_2_, diamide and menadione induces extensive modification of cellular proteins by CoA in examined total protein extracts. Weak background immunoreactive signal was observed in control untreated cells. Immunoblotting of pulled down SBP-Prdx5 revealed strong induction of Prdx5 CoAlation in cells treated with all oxidising agents, and the highest level is observed in response to diamide treatment (Fig. 2b).

To further validate the above findings, we examined CoAlation of endogenous Prdx5 from HEK293/Pank1β cells or primary rat cardiomyocytes treated or untreated with 500 μM diamide for 30 min. Endogenous Prdx5 was immunoprecipitated from total cell lysates with anti-Prdx5 antibody and the immune complexes probed with anti-CoA and anti-Prdx5 antibodies. The induction of endogenous Prdx5 CoAlation in response to diamide treatment is shown in Fig. 2c, d.

### Prdx5 CoAlation is induced by glucose deprivation

Glucose is the primary source of energy in most eukaryotic cells and glucose deprivation causes metabolic oxidative stress. The observation that culturing HEK293/Pank1β cells in pyruvate-free and low glucose medium resulted in subtle increase of CoAlated Prdx5 (Fig. 2c), led us to examine the effect of metabolic stress induced by glucose deprivation on Prdx5 CoAlation. Here, HEK293/Pank1β cells were transfected with pCR3.1-MAR/wt-Prdx5 plasmid and, 24 h later, the transfection medium was replaced with pyruvate- and glucose-free DMEM. Cells were harvested after 18 h and total protein extracts and streptavidin-Sepharose pulled down proteins were analysed by immunoblotting with anti-CoA antibody.

**Fig. 3:**
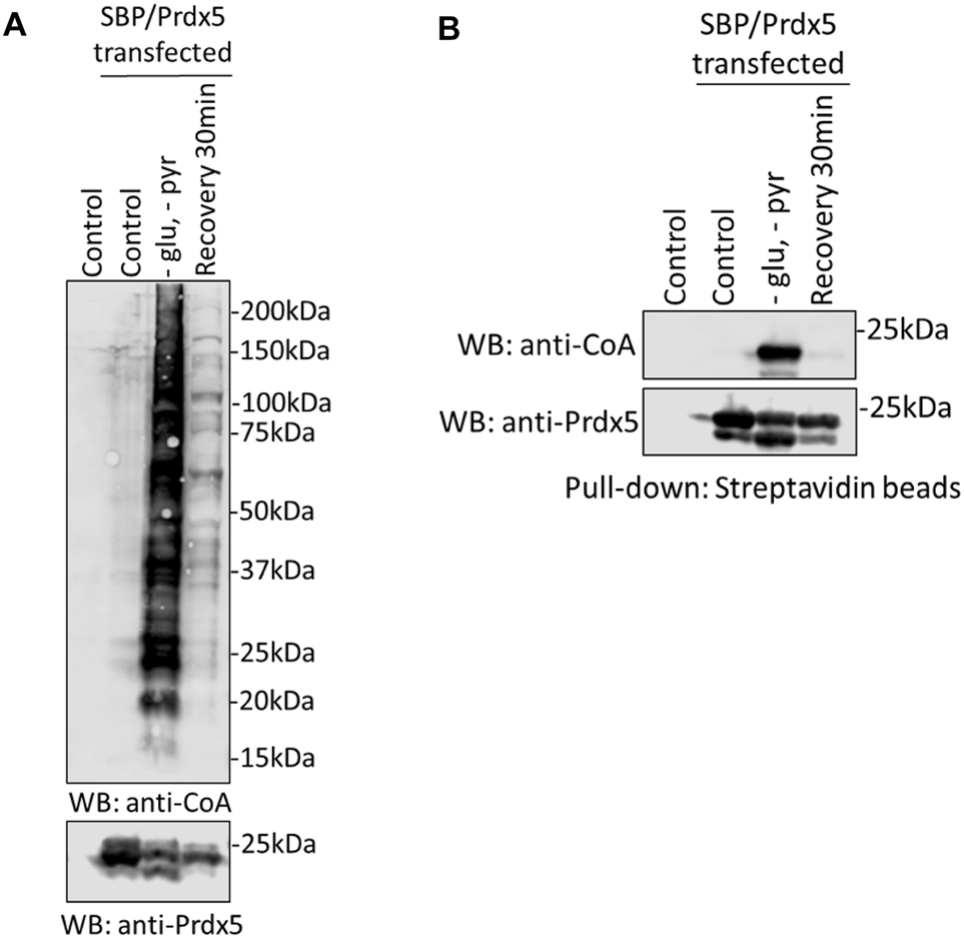
CoAlation of transiently overexpressed Prdx5 in HEK293/Pank1β cells is induced by metabolic stress. HEK293/Pank1β cells were grown in pyruvate and glucose-free media for 18 h to induce metabolic stress. To find whether Prdx5 CoAlation is a reversible post-translational modification, complete media was then re-introduced for 30 min (recovery lane). Total protein (**a**) or SBP-Prdx5 (**b**) CoAlation was analysed by anti-CoA immunoblot. N = 3

As shown in Fig. 3a, significant increase in protein CoAlation was observed in cells cultured in glucose-deprived media. Notably, adding full media back to metabolically stressed cells for 30 min resulted in significant de-CoAlation of cellular proteins, which was almost at the level of control cells. Immunoblotting of streptavidin-Sepharose pull-down complexes with anti-CoA antibodies revealed markedly increased CoAlation of SBP-Prdx5 in glucose-deprived cells (Fig. 3b). A background level of SBP-Prdx5 CoAlation was observed in control cells. To find out whether the induction of SBP-Prdx5 CoAlation by glucose deprivation is a reversible post-translational modification, complete media was re-introduced for 30 min. As shown in Fig. 3b, glucose deprivation-induced Prdx5 CoAlation is completely reversed after the re-addition of complete DMEM medium.

### Mutational analysis of Prdx5 CoAlation in response to oxidative and metabolic stress

**Fig. 4:**
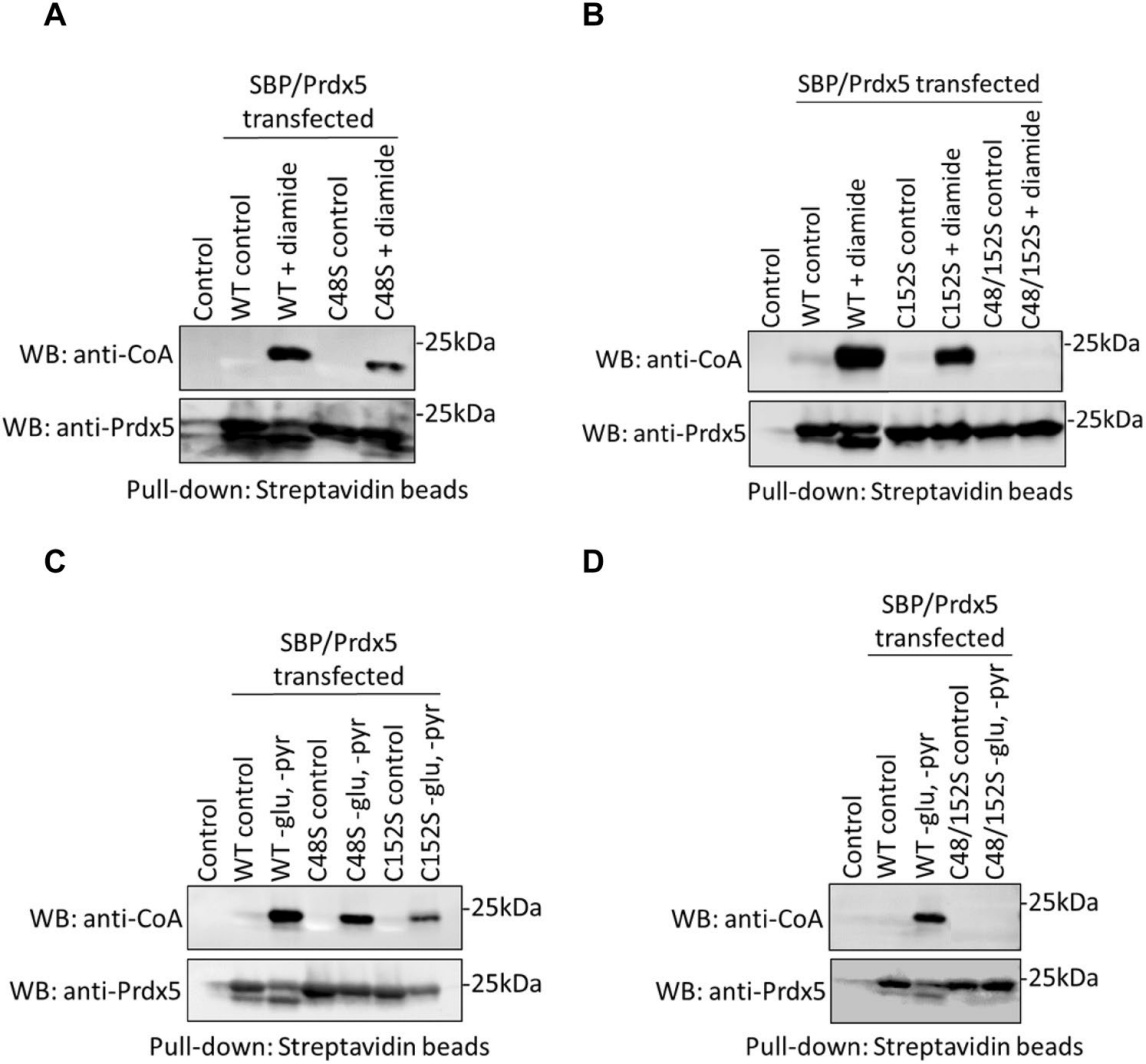
Mutational analysis of Prdx5 CoAlation in cellular response to oxidative and metabolic stress. **a** HEK293/Pank1β cells transfected with wild type (WT) and C48S mutant of SBP-Prdx5 were incubated with and without 500 µM diamide for 30 min; **b** HEK293/Pank1β cells transfected with wild type, C152S and C48/152S mutants of SBP-Prdx5 were incubated with or without 500 µM diamide for 30 min. **c**, **d** HEK293/Pank1β cells transfected with wild type and mutants of SBP-Prdx5 were grown in pyruvate (pyr) and glucose (glu) free media for 18 h to induce metabolic stress. Transiently overexpressed SBP-tagged WT Prdx5, C48S, C152S and C48/152S mutants were pulled down with Streptavidin beads and examined by anti-CoA immunoblot. N = 3

Prdx5 possesses three cysteine residues: Cys48 and Cys152 correspond to peroxidatic and resolving cysteines respectively, while Cys73 is not involved in the catalytic mechanism [23]. The LC–MS/MS analysis revealed CoAlation of peroxidatic Cys48 in HEK293/Pank1β cells and perfused rat heart exposed to H_2_O_2_. To validate this finding and investigate whether other cysteine residues are covalently modified by CoA under oxidative and metabolic stress, we carried out mutational studies using a panel of hPrdx5 mutants (C48S, C152S or C48/152S). Initially, we examined at which site/sites Prdx5 is CoAlated under oxidative stress. Here, HEK293/Pank1β cells were transfected with pCR3.1-MAR plasmids, expressing wild type or generated mutants of Prdx5. 24 h after transfection, cells were incubated for 30 min with or without 500 µM diamide. Streptavidin pull-downs from lysed cells were analysed by Western blotting with anti-CoA and anti-Prdx5 antibodies. The anti-Prdx5 blot shows a comparable amount of pulled down SBP-Prdx5 in all transfected cells. Diamide-induced CoAlation of wtPrdx5 is readily detected in anti-CoA blot of streptavidin pull-downs (Fig. 4a and b). The C48S and C152S mutants were also CoAlated, but at a reduced level, suggesting CoAlation of both peroxidatic and resolving cysteines in cellular response to oxidative stress. This assumption was confirmed when we examined diamide-induced CoAlation of the C48/152S double mutant. As shown in Fig. 4b, the C48/152S double mutant is not CoAlated in control and diamide-treated cells. These findings also indicate that the non-catalytic Cys73 is not CoAlated in cells exposed to oxidising agents.

We also investigated CoAlation of transiently overexpressed wild type and cysteine mutants of SBP-Prdx5 in HEK293/Pank1β cells under metabolic stress induced by glucose deprivation. Immunoblotting of streptavidin-Sepharose pulled down proteins with anti-CoA antibody showed background CoAlation in control cells (Fig. 4c and d). As expected, significant CoAlation of transiently expressed wtPrdx5 was detected in glucose-deprived cells. Similar to diamide-treated cells, CoAlation of the C48S and C152S mutants was significantly lower, when compared to wtPrdx5 (Fig. 4c and d). No CoAlation of the C48/152S double mutant was observed in glucose-deprived cells.

### Peroxidase activity of Prdx5 is inhibited by covalent binding of CoA in vitro

We have recently developed an in vitro CoAlation assay, which allows covalent CoA modification of purified recombinant and endogenous proteins with high efficiency [17]. Using this assay, we showed that in vitro CoAlation of several enzymes from different metabolic pathways, including creatine kinase (CK) and glyceraldehyde-3-phosphate dehydrogenase (GAPDH), results in significant inhibition of their enzymatic activities [17].

**Fig. 5:**
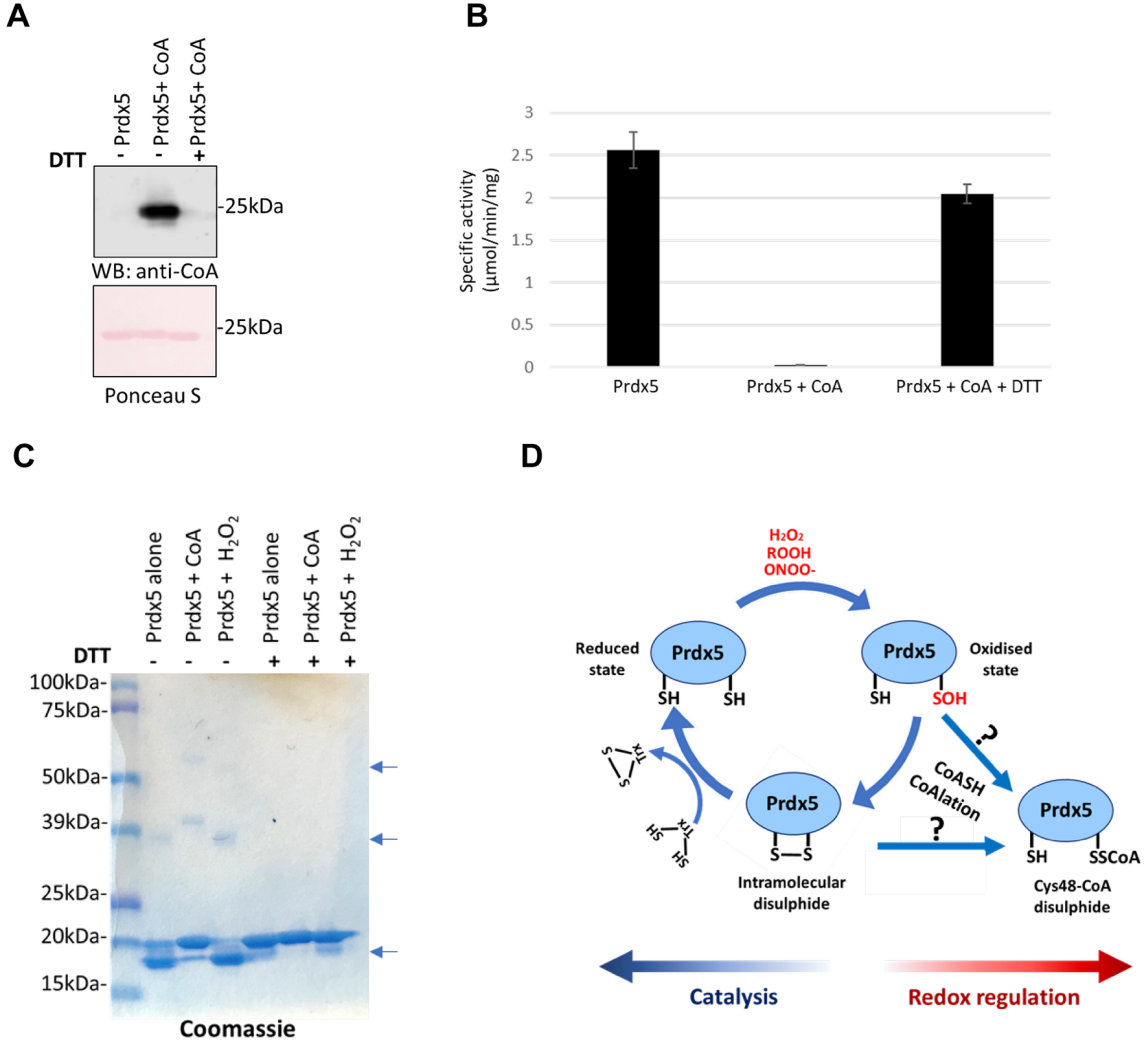
Regulation of the Prdx5 enzymatic cycle by CoAlation **a** In vitro CoAlted-Prdx5 was boiled in loading buffer with or without DTT. CoAlation of Prdx5 was examined by anti-CoA immunoblot. **b** NADPH oxidation coupled to the peroxidase activity of Prdx5 in the presence of the Trx systems. The initial rate of NADPH oxidation was monitored by measurement of the decrease in *A*_340_ in the presence of Prdx5 at 37 °C. The 150 μl reaction mixture contained 50 mM Hepes–NaOH (pH 7), 200 μM NADPH, 76 nM mouse TrxR, 1.1 μM human Trx, and 2 μg untreated Prdx5 (control), in vitro CoAlated Prdx5 or in vitro CoAlated Prdx5 with DTT. The reaction was initiated by the addition of 0.5 mM H_2_O_2_. **c** Recombinant Prdx5 was incubated with oxidised and reduced CoA or with H_2_O_2_ for 30 min, or with buffer (Prdx5 alone). The samples were separated by SDS-PAGE under reducing (+DTT), and non-reducing conditions (-DTT). The arrows indicate the position of monomeric and dimeric forms of His-Prdx5. **d** Schematic representation of the Prdx5 catalytic cycle. In the presence of a substrate molecule, Prdx5 peroxidatic cysteine is oxidised to sulphenic acid (Cys-SOH) and condenses to form an intramolecular disulphide with the resolving cysteine. In prolonged oxidative or metabolic stress conditions, the CoA thiol attacks the cysteine sulphenic acid, or disulphide bond and binds to catalytic Cys48

To test the effect of CoAlation on Prdx5 peroxidase activity, recombinant His-Prdx5 was incubated with or without a mix of CoASH (reduced) and CoASSCoA (oxidised) in the CoAlation buffer for 30 min. Then, the reaction mixture was passed through a BioSpin column to remove the excess of reduced and oxidised CoA. The efficiency of in vitro CoAlation was confirmed by Western blotting with anti-CoA antibody. As shown on Fig. 5a, recombinant His-Prdx5 was modified by CoA in vitro in a DTT-sensitive manner. Prdx5 activity towards H_2_O_2_ was then assayed by measuring the decrease in NADPH absorbance at 340 nm as described in M&M. Figure 5b demonstrates that in vitro CoAlation of His-Prdx5 results in near complete inhibition (98.7%) of the peroxidase activity. Furthermore, the inhibitory effect of CoAlation on Prdx5 activity was efficiently reversed by the addition of 100 mM DTT to the reaction mix. These data indicate that covalent modification of catalytic cysteines by CoA results in the inhibition of Prdx5 peroxidase activity and the inhibitory effect is reversed by the reducing power of DTT.

We also examined electrophoretic mobility of control (untreated), oxidised (H_2_O_2_ treated) and CoAlated His-Prdx5 separated under reducing and non-reducing conditions. This analysis showed that control, oxidised and CoAlated His-Prdx5 run as a single band of ~ 20 kDa under reducing conditions (Fig. 5c). However, different pattern of electrophoretic mobility was observed under non-reducing conditions. In vitro CoAlated Prdx5 showed the same mobility under reducing and non-reducing conditions (~ 20 kDa). The exposure of recombinant His-Prdx5 to H_2_O_2_ results in faster mobility (~ 18 kDa) under non-reducing conditions, suggesting significant conformational changes in a monomeric form of Prdx5 mediated by a disulphide bond formation. These findings are in agreement with published crystallographic studies of reduced and oxidised forms of Prdx5 [23, 24]. The distance between the two sulphur atoms of peroxidatic Cys48 and resolving Cys152 in a reduced form of Prdx5 is 13.8 A° [24]. The overall fold of oxidised Prdx5 is very similar to that of the reduced form and the main conformational changes involve the movement of the regions containing peroxidatic and resolving cysteines, allowing the formation of a disulphide bridge and a more compact fold [23]. A significant proportion of control His-Prdx5, which was stored at − 20 °C in buffer without DTT, also runs with higher electrophoretic mobility. These data indicate that recombinant Prdx5 is prone to oxidation when stored without DTT even for a short period of time. It has been reported that freshly grown Prdx5 crystals can only survive for a few days when they are simply exposed to air, whereas crystals of the C48S mutant are much more stable, even in presence of H_2_O_2_ [24].

It has been reported that PRDX5 can also form dimers, known as type A dimers, and most contacts involve hydrophobic interactions with the exception of an inter-molecular salt bridge between Arg124 and Asp77 [23]. A minor percentage of dimeric (~ 40 kDa) and potentially trimeric (~ 60 kDa) forms of Prdx5 was observed under non-reducing conditions, and their appearance is DTT-dependent (Fig. 5c).

## Discussion

Prdx5 was the last mammalian isoform of the Prdx family to be identified and therefore, the knowledge on its regulation by signalling pathways via binding partners and post-translational modifications is lagging. To date, several post-translational modifications of Prdx5, including phosphorylation, acetylation and glutathionylation, have been reported in proteome-wide studies, but their physiological relevance remains unclear [27–29]. Interestingly, glutathionylation of Prdx5 was detected by MALDI-MS in primary rat hepatocytes exposed to oxidative stress. However, the site of glutathionylation and significance of this modification in the regulation of Prdx5 catalytic activity has yet to be investigated [28].

In this paper, we report for the first-time covalent modification of Prdx5 by CoA in cellular response to oxidative and metabolic stress. Evidence is provided that Prdx5 is CoAlated at low background level in exponentially growing cells, while exposure to oxidising agents induces covalent modification of transiently overexpressed and endogenous Prdx5 by CoA. Furthermore, metabolic stress induced by glucose deprivation also leads to a significant increase in Prdx5 CoAlation, which is efficiently reversed by the re-addition of glucose to the culture medium. These findings are in agreement with our recently published studies on redox regulation of protein CoAlation in prokaryotic and eukaryotic cells [17, 18].

Human Prdx5 and orthologues in vertebrates and invertebrates possess three highly conserved cysteine residues: Cys48, Cys73 and Cys152. Crystallographic studies of hPrdx5 in reduced and oxidised states revealed that both peroxidatic Cys48 and resolving Cys152 are surface exposed, while Cys73 is completely buried [23, 24]. The LC–MS/MS analysis of CoAlated proteins in HEK293/Pank1β cells and perfused rat hearts exposed to H_2_O_2_ revealed CoAlation of only the peroxidatic Cys48. The peroxidatic cysteines are more nucleophilic and highly sensitive to attack by reactive oxygen and reactive nitrogen species, and therefore are more prone to regulatory S-thiolation, than resolving cysteine residues. The peroxidatic Cys48 in hPrdx5 has a low pKa (5.2), making it deprotonated at physiological pH and more nucleophilic [29]. In normal metabolic conditions, the peroxidatic cysteine residue of Prdx5 reduces a peroxide substrate and is oxidised to sulphenic acid. The resolving Cys152 then attacks this cysteine sulphenic acid forming an intramolecular disulphide bond, which is reduced by thioredoxin to regenerate an active enzyme. Thioredoxin reductase reduces the oxidised thioredoxin, using NADPH as a reductant. In conditions of severe oxidative stress or prolonged metabolic stress, NADPH is depleted and therefore the NADPH-dependent recycling system for Prdx5 might not function efficiently to reduce the intramolecular disulphide bond of oxidised Prdx5. We hypothesise that the CoA thiol attacks the Cys48 cysteine sulphenic acid, or the disulphide bond formed between the peroxidatic Cys48 and the resolving Cys152, and binds to protect the peroxidatic Cys48 from overoxidation. Indeed, it was shown in previous studies that treatment of cells with 100–200 µM peroxides (t-butyl hydroperoxide, H_2_O_2_) is sufficient to cause overoxidation of Prdxs [30–32]. CoAlation of Prdx5 might be a regulatory event to protect the catalytic Cys48 from irreversible overoxidation. In mutational studies we observed oxidative and metabolic stress-induced CoAlation of both peroxidatic Cys48 and resolving Cys152. We speculate that in wild type Prdx5, peroxidatic Cys48 is a preferred target for the oxidation-mediated covalent modification by CoA, while CoAlation of resolving Cys152 is a specific attribute of the Prdx5C48S mutant. Covalent modification of peroxidatic Cys48 by CoA implies the inhibitory effect of CoAlation on Prdx5 peroxidase activity. Indeed, in vitro CoAlated recombinant Prdx5 was found to be enzymatically inactive and the peroxidase activity was recovered by DTT-mediated dissociation of CoA. Our studies in metabolic stress showed that CoAlation of Prdx5 is physiologically reversible, and we have proposed the existence of CoA-redoxins that would act to reduce CoA-modified proteins [33].

These results are in agreement with our previous studies, in which we demonstrated that in vitro CoAlation of catalytically active cysteines in several metabolic enzymes, including CK, pyruvate dehydrogenase kinase 2 (PDK2) and GAPDH, impedes their catalytic activities and the inhibition is DTT-sensitive [17]. Furthermore, we have also demonstrated that in vitro CoAlation of *Staphylococcus aureus* GAPDH not only inhibits the enzymatic activity, but also protects the catalytic Cys151 from overoxidation by H_2_O_2_ [18]. The question which remains to be answered is whether redox-induced Prdx5 CoAlation serves to protect catalytic cysteines from overoxidation and to upregulate the antioxidant defence via redox signalling. Recently, glutathionylation of Prdx2 under oxidative stress conditions was shown to protect the catalytic cysteines from hyperoxidation and play a role in redox signalling [34, 35].

The attachment of pantetheine and 3′5′-ADP moieties to CoA-modified cysteines in oxidative stress response may generate a unique binding motif for intra- and inter-molecular interactions, especially for proteins containing the nucleotide binding fold. It has been recently reported that redox-mediated modification of Prdx1 by GSH induces its interaction with phosphatase and tensin homolog (PTEN) and mammalian Ste20-like kinase-1 (MST1) in the regulation of pro-survival signalling and the cell cycle respectively [36, 37]. We speculate that Prdx5 CoAlation in cellular response to oxidative and metabolic stress may promote the formation or dissociation of regulatory complexes, which are involved in redox signalling and antioxidant defence. Recently, specific interaction between superoxide dismutase 1 (SOD1) and Prdx5 was shown to be critical for maintaining mitochondrial redox homeostasis and avoiding cell death [38]. It will be interesting to investigate whether the SOD1/Prdx5 interaction is affected by covalent modification of Prdx5 catalytic cysteines in cellular response to oxidative and metabolic stress.

Taken together, the findings presented in this study permit us to propose that the Prdx5 catalytic cycle operates when permissible levels of H_2_O_2_ are produced in cells under oxidative stress (Fig. 5c). Further accumulation of H_2_O_2_ can induce Prdx5 CoAlation, which may function to protect catalytic cysteines from overoxidation and initiate redox signalling pathways involved in antioxidant defences and the repair of oxidative damage.

## Abbreviations

PRDX5: Peroxiredoxin 5
SBP: Streptavidin binding protein
LC–MS/MS: Liquid chromatography tandem-mass spectrometry

## References

[1] Rhee SG, Kil IS (2017). Multiple functions and regulation of mammalian peroxiredoxins. Annu Rev Biochem.

[2] Perkins A, Nelson KJ, Parsonage D (2015). Peroxiredoxins: guardians against oxidative stress and modulators of peroxide signaling. Trends Biochem Sci.

[3] Fischer F, Leipelt M, Wolters D, Steegborn C (2009). Identification of Peroxiredoxin 1 as a novel interaction partner for the lifespan regulator protein p66Shc. Aging (Albany NY).

[4] Kang DH, Lee DJ, Lee S (2017). Interaction of tankyrase and peroxiredoxin II is indispensable for the survival of colorectal cancer cells. Nat Commun.

[5] Sorokina EM, Feinstein SI, Zhou S (2011). Intracellular targeting of peroxiredoxin 6 to lysosomal organelles requires MAPK activity and binding to 14-3-3 ε. Am J Physiol Cell Physiol.

[6] Nguyên-nhu NT, Berck J, Clippe A (2007). Human peroxiredoxin 5 gene organization, initial characterization of its promoter and identification of alternative forms of mRNA. Biochim Biophys Acta Gene Struct Expr.

[7] Knoops B, Goemaere J, Van der Eecken V, Declercq JP (2011). Peroxiredoxin 5: structure, mechanism, and function of the mammalian atypical 2-cys peroxiredoxin. Antioxid Redox Signal.

[8] Barranco-Medina S, Lázaro JJ, Dietz KJ (2009). The oligomeric conformation of peroxiredoxins links redox state to function. FEBS Lett.

[9] Radyuk SN, Michalak K, Klichko VI (2009). Peroxiredoxin 5 confers protection against oxidative stress and apoptosis and also promotes longevity in Drosophila. Biochem J.

[10] Leonardi R, Zhang YM, Rock CO, Jackowski S (2005). Coenzyme A: back in action. Prog Lipid Res.

[11] Davaapil H, Tsuchiya Y, Gout I (2014). Signalling functions of coenzyme A and its derivatives in mammalian cells. Biochem Soc Trans.

[12] Srinivasan B, Sibon OCM (2014). Coenzyme A, more than “just” a metabolic cofactor. Biochem Soc Trans.

[13] Theodoulou FL, Sibon OCM, Jackowski S, Gout I (2014). Coenzyme A and its derivatives: renaissance of a textbook classic. Biochem Soc Trans.

[14] Mcallister RA, Fixter LM, Campbell EHG (1988). The effect of tumour growth on liver pantothenate, CoA, and fatty acid synthetase activity in the mouse. Br J Cancer.

[15] Brass EP, Tahiliani AG, Allen RH, Stabler SP (1990). Coenzyme a metabolism in vitamin B-12—deficient rats. J Nutr.

[16] Reibel DK, Wyse BW, Berkich DA, Neely JR (1981). Regulation of coenzyme A synthesis effects of diabetes and fasting in heart muscle: effects of diabetes and fasting. Am J Physiol.

[17] Tsuchiya Y, Peak-Chew SY, Newell C (2017). Protein CoAlation: a redox-regulated protein modification by coenzyme A in mammalian cells. Biochem J.

[18] Tsuchiya Y, Zhyvoloup A, Baković J (2018). Protein CoAlation and antioxidant function of coenzyme A in prokaryotic cells. Biochem J.

[19] Malanchuk OM, Panasyuk GG, Serbin NM (2015). Generation and characterization of monoclonal antibodies specific to coenzyme A. Biopolym Cell.

[20] Chouchani ET, Pell VR, Gaude E (2014). Ischaemic accumulation of succinate controls reperfusion injury through mitochondrial ROS. Nature.

[21] Choi HI, Chung KJ, Yang HY (2013). Peroxiredoxin v selectively regulates IL-6 production by modulating the Jak2-Stat5 pathway. Free Radic Biol Med.

[22] Clark H, Carling D, Saggerson D (2004). Covalent activation of heart AMP-activated protein kinase in response to physiological concentrations of long-chain fatty acids. Eur J Biochem.

[23] Smeets A, Marchand C, Linard D, Knoops B, Declercq JP (2008). The crystal structures of oxidized forms of human peroxiredoxin 5 with an intramolecular disulfide bond confirm the proposed enzymatic mechanism for atypical 2-cys peroxiredoxins. Arch Biochem Biophys.

[24] Seo MS, Kang SW, Kim K (2000). Identification of a new type of mammalian peroxiredoxin that forms an intramolecular disulfide as a reaction intermediate. J Biol Chem.

[25] Cox J, Mann M (2008). MaxQuant enables high peptide identification rates, individualized p.p.b.-range mass accuracies and proteome-wide protein quantification. Nat Biotechnol.

[26] Tsuchiya Y, Peak-Chew SY, Newell C (2017). Protein CoAlation: a redox-regulated protein modification by coenzyme A in mammalian cells. Biochem J.

[27] Nguyen TTM, Wong R, Menazza S (2014). Cyclophilin D modulates the mitochondrial acetylome Tiffany. Circ Res.

[28] Fratelli M, Demol H, Puype M (2003). Identification of proteins undergoing glutathionylation in oxidatively stressed hepatocytes and hepatoma cells. Proteomics.

[29] Zemskova M, Ling J, Lilly M (2007). PIM1 kinase protects cells from death induced by oxidative stress: potential role of PRDX5 phosphorylation. Cancer Res.

[30] Yang KS, Kang SW, Woo HA (2002). Inactivation of human peroxiredoxin I during catalysis as the result of the oxidation of the catalytic site cysteine to cysteine-sulfinic acid. J Biol Chem.

[31] Rabilloud T, Heller M, Gasnier F (2002). Proteomics analysis of cellular response to oxidative stress. evidence for in vivo overoxidation of peroxiredoxins at their active site. J Biol Chem.

[32] So YK, Jo HY, Mi HK (2008). H_2_O_2_-dependent hyperoxidation of peroxiredoxin 6 (Prdx6) plays a role in cellular toxicity via up-regulation of iPLA2 activity. J Biol Chem.

[33] Gout I (2018). Coenzyme A, protein CoAlation and redox regulation in mammalian cells. Biochem Soc Trans.

[34] Peskin AV, Pace PE, Behring JB (2016). Glutathionylation of the active site cysteines of peroxiredoxin 2 and recycling by glutaredoxin. J Biol Chem.

[35] Netto LES, Antunes F (2016). The roles of peroxiredoxin and thioredoxin in hydrogen peroxide sensing and in signal transduction. Mol Cells.

[36] Morinaka A, Funato Y, Uesugi K, Miki H (2011). Oligomeric peroxiredoxin-I is an essential intermediate for p53 to activate MST1 kinase and apoptosis. Oncogene.

[37] Cao J, Schulte J, Knight A (2009). Prdx1 inhibits tumorigenesis via regulating PTEN/AKT activity. EMBO J.

[38] Malty RH, Aoki H, Kumar A (2017). A map of human mitochondrial protein interactions linked to neurodegeneration reveals new mechanisms of redox homeostasis and NF-κB signaling. Cell Syst.

